# Combined Analysis of Variation in Core, Accessory and Regulatory Genome Regions Provides a Super-Resolution View into the Evolution of Bacterial Populations

**DOI:** 10.1371/journal.pgen.1006280

**Published:** 2016-09-12

**Authors:** Alan McNally, Yaara Oren, Darren Kelly, Ben Pascoe, Steven Dunn, Tristan Sreecharan, Minna Vehkala, Niko Välimäki, Michael B. Prentice, Amgad Ashour, Oren Avram, Tal Pupko, Ulrich Dobrindt, Ivan Literak, Sebastian Guenther, Katharina Schaufler, Lothar H. Wieler, Zong Zhiyong, Samuel K. Sheppard, James O. McInerney, Jukka Corander

**Affiliations:** 1 Pathogen Research Group, Nottingham Trent University, Nottingham, United Kingdom; 2 Institute of Microbiology and Infection, University of Birmingham, Birmingham, United Kingdom; 3 Department of Cell Research and Immunology, George S. Wise Faculty of Life Sciences, Tel Aviv University, Tel Aviv, Israel; 4 Department of Biology, National University Ireland, Maynooth, Ireland; 5 College of Medicine, University of Swansea, Swansea, United Kingdom; 6 Department of Mathematics and Statistics, University of Helsinki, Helsinki, Finland; 7 Departments of Pathology and Microbiology, University College Cork, Cork, Ireland; 8 Institute of Hygiene, Universitat Muenster, Muenster, Germany; 9 Department of Biology and Wildlife Diseases, Faculty of Veterinary Hygiene and Ecology, and CEITEC VFU, University of Veterinary and Pharmaceutical Sciences, Brno, Czech Republic; 10 Centre for Infection Medicine, Institute of Microbiology and Epizootics, Freie Universitat, Berlin, Germany; 11 Robert Koch Institute, Berlin, Germany; 12 Centre for Infectious Diseases, West China Hospital of Sichuan University, Chengdu, China; 13 Faculty of Life Sciences, The University of Manchester, Manchester, United Kingdom; 14 Department of Biostatistics, University of Oslo, Oslo, Norway; Uppsala University, SWEDEN

## Abstract

The use of whole-genome phylogenetic analysis has revolutionized our understanding of the evolution and spread of many important bacterial pathogens due to the high resolution view it provides. However, the majority of such analyses do not consider the potential role of accessory genes when inferring evolutionary trajectories. Moreover, the recently discovered importance of the switching of gene regulatory elements suggests that an exhaustive analysis, combining information from core and accessory genes with regulatory elements could provide unparalleled detail of the evolution of a bacterial population. Here we demonstrate this principle by applying it to a worldwide multi-host sample of the important pathogenic *E*. *coli* lineage ST131. Our approach reveals the existence of multiple circulating subtypes of the major drug–resistant clade of ST131 and provides the first ever population level evidence of core genome substitutions in gene regulatory regions associated with the acquisition and maintenance of different accessory genome elements.

## Author Summary

We present an approach to evolutionary analysis of bacterial pathogens combining core genome, accessory genome, and gene regulatory region analyses. This enables unparalleled resolution of the evolution of a multi-drug resistant pandemic pathogen that would remain invisible to a core genome phylogenetic analysis alone. In particular, our combined analysis approach identifies population-level evidence for compensatory mutations offsetting the costs of resistance plasmid maintenance as a key event in the emergence of dominant MDR lineages of *E*. *coli*.

## Introduction

The ability to sequence hundreds or thousands of bacteria genomes in a timely and cost effective manner has allowed microbiologists to study microbial evolution at an unprecedented scale and level of resolution [1]. The focus of microbial population genomics research has often involved the creation of core genome phylogenetic trees to reconstruct the evolutionary trajectory of a pathogenic species or subspecies. Core genomes can be obtained by mapping genome data against a common reference sequence or by extracting the coding sequences (CDS) which are common across all members of a given data set. This approach has led to significant discoveries of the emergence of pathogenic bacteria [2–5], and allows fine scale analysis to inform interpretation of transmission events [6,7].

Whilst the use of core genome phylogenetics has improved understanding of the evolution of bacterial pathogens, discarding information from genes which are differentially present in a bacterial population (the accessory genome) results in the loss of a large amount of potentially useful genetic information. Pan genome analyses of bacteria from the Enterobacteriaceae show that the core genome accounts for only a small proportion of the entire gene pool of a species [8,9]. Integrating accessory gene pool analysis can improve the resolution of core genome phylogenetic studies. For example, investigation of the accessory genome of *Yersinia enterocolitica* revealed ecological patterns of separation within the population [9] and a study of a global collection of enterotoxigenic *Escherichia coli* (ETEC) used plasmid profiling to identify multiple clones of ETEC circulating globally [10]. Furthermore a study of the accessory genome of *Klebsiella pneumoniae* identified virulence loci significantly associated with isolates from invasive disease [11].

The acquisition of lineage specific gene-regulatory regions in the core genome has also been recently shown to play a key role in the formation of phylogenetically distinct phenotypes in *E*. *coli* [12]. Therefore, systematic analysis of sequence variation in regulatory regions should complement the information provided by the core and accessory CDSs. In this study we analyse the core and accessory genome jointly with core regulatory elements to provide unprecedented insight into the population structure and ecological inference of the globally important human pathogen *E*. *coli* ST131. This lineage of extra-intestinal pathogenic *E*. *coli* (ExPEC) has been rapidly globally disseminated to become the dominant multi-drug resistant (MDR) isolate of *E*. *coli* from urinary tract and bloodstream infections across the world [13]. Three distinct clades have been identified within the ST131 lineage [14,15], of which clade C, also known as H30Rx, is associated with the rapid expansion and global dissemination of MDR isolates carrying the CTX-M-15 extended spectrum beta lactamase (ESBL). However, ambiguities remain regarding the importance of some undersampled reservoirs of resistant strains [16, 17, 18, 19]. First, while frequent plasmid movement between poultry and human ST-131 isolates and clustering of marine animal isolates with those of human [15], suggests ecological overlap of human and animal niches, human and agricultural animal strains are rarely isolated from the same environment [20]. Second, diverse sets of human clinical isolates show distinct plasmids associated with each CTX-M type, highlighting the importance of sampling isolates from people in multiple countries.

Here we study the emergence of this important human pathogen by analysing the genomes of diverse isolates from avian species, domesticated animals, and humans encompassing a full spectrum of geographical and ESBL gene diversity. Our combined analysis allows the highest resolution view to date of the population structure of the ST131 lineage and shows how emergence of distinct MDR clusters are underpinned by associated changes in the core gene regulatory regions.

## Results

### Broad host range of *E*. *coli* ST131

**Fig 1:**
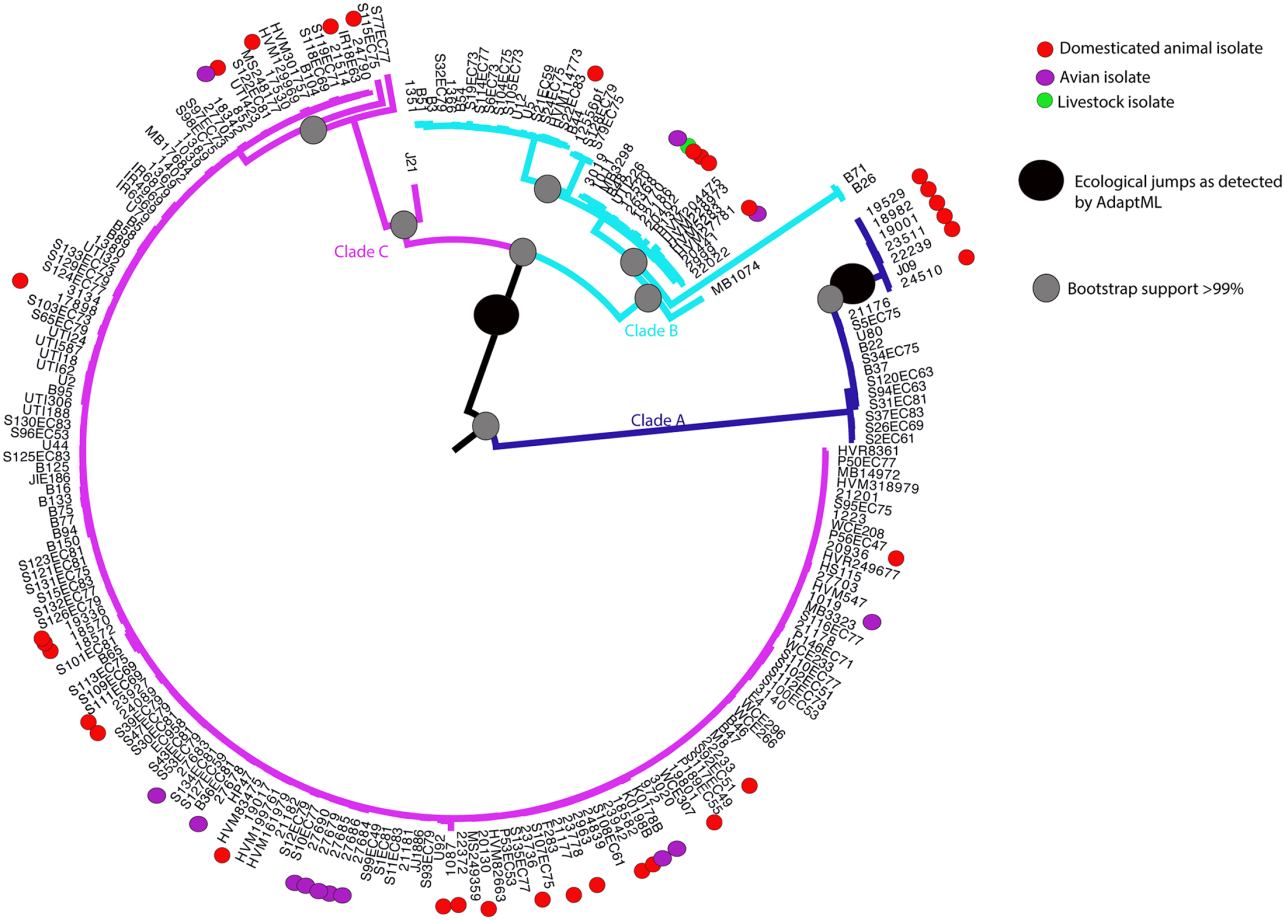
Maximum likelihood phylogeny of 228 *E*. *coli* ST131 isolates. Strains isolated from dogs and cats (domesticated animals), wild birds (avian), and cattle (livestock) are indicated by colour coding at the tips of the tree, with all other strains not colour coded being human isolates. Clades A, B and C are indicated by colour coding of the branches. The large black circles indicate statistically significant inferences of host jumps or ecological adaptations within the phylogeny as detected by AdaptML. The grey circles indicate phylogenetic inferences with > 99% bootstrap support. The names of the taxa match those in S1 Table.

We used a total of 228 *E*. *coli* ST131 genome sequences (S1 Table). Of these 125 were avian, domesticated animal, and human clinical isolates with a broad range of CTX-M gene type sequenced as part of this study, and the remaining 103 were human clinical isolates from previous phylogenomic studies. A core-genome alignment and maximum likelihood phylogeny was obtained from localised co-linear blocks (length = 3,749,897bp) for all 228 genomes which revealed a 3-clade structure identical to those previously described (Fig 1). In total, 16,799 SNPs were present in the alignment, with 3,985 SNPs present in clade C, in agreement with previous core genome phylogenetic studies [14,15]. Isolates from wild birds, cats and dogs were distributed throughout the phylogenetic tree suggesting frequent cross-species movement of strains, with the exception of a group of predominantly domesticated animal isolates in clade A, and a small group of avian isolates in clade C. AdaptML analysis of the phylogeny confirmed the lack of any clear host or ecological boundaries within the phylogeny, identifying just two significant host jump events in the population. The first is in the shift from clade A to clade B/C, whilst another event is indicated in the shift from small sub-cluster in clade A to the remainder of the phylogeny (Fig 1). This suggests that *E*. *coli* ST131 is a host generalist pathogen capable of frequent inter-species movement.

### Multiple *E*. *coli* ST131 clusters based on accessory genome analysis

**Fig 2:**
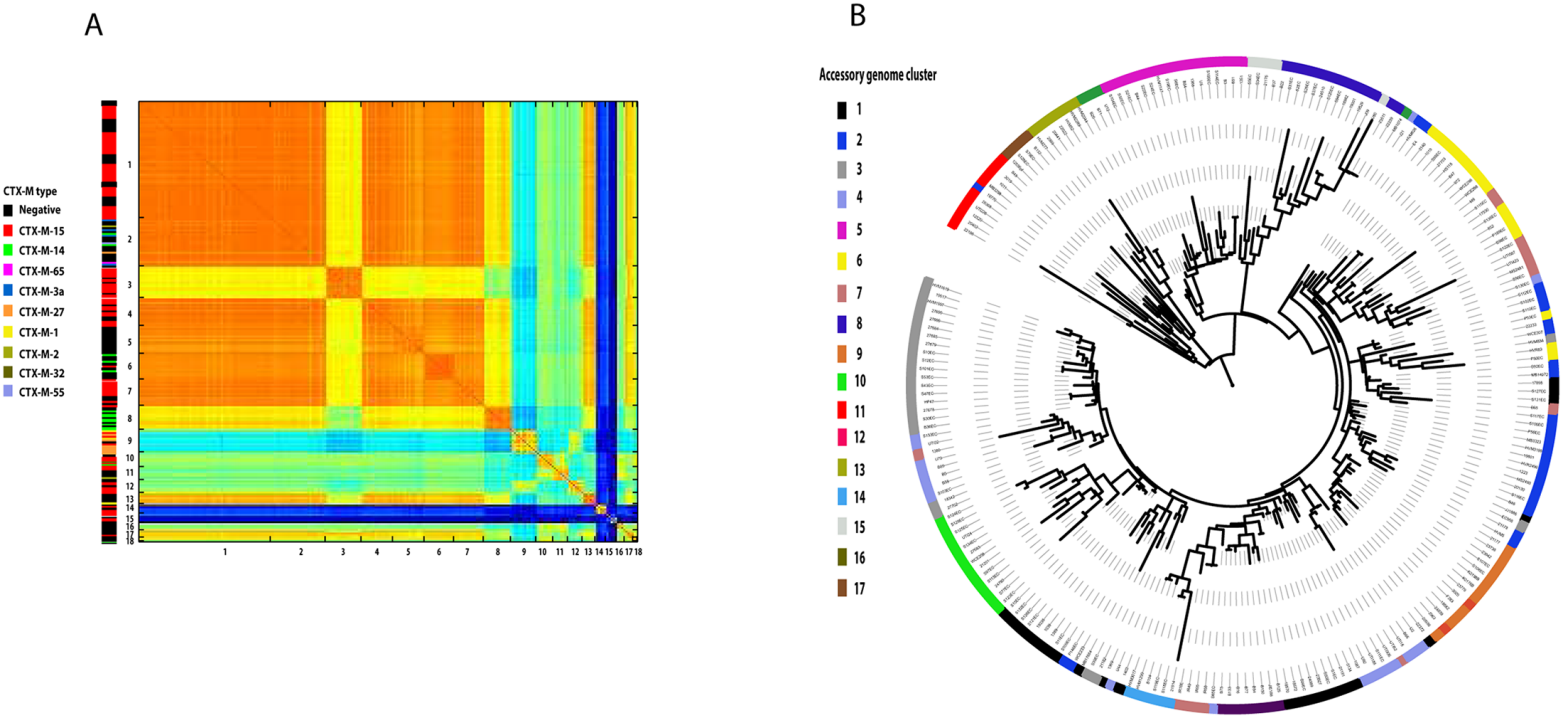
A) Graphical representation of the clustering of isolates based on their accessory gene content based on a pairwise comparison of the accessory gene content of all 228 genomes. The colour scheme is a heatmap representation of the levels of identity of accessory genes between strains based on the BLAST score output from LS-BSR, with red equalling 100% and dark blue representing 0%. Numbers on the X and Y-axis indicate the accessory genome cluster labels. The ESBL gene type of each strain is indicated by the colour coded bar on the Y-axis. B) A maximum likelihood phylogenetic tree of the accessory genome of all isolates based on a binary gene presence-v-absence alignment file. Colour coding refers to the accessory genome clusters identified in panel A.

**Fig 3:**
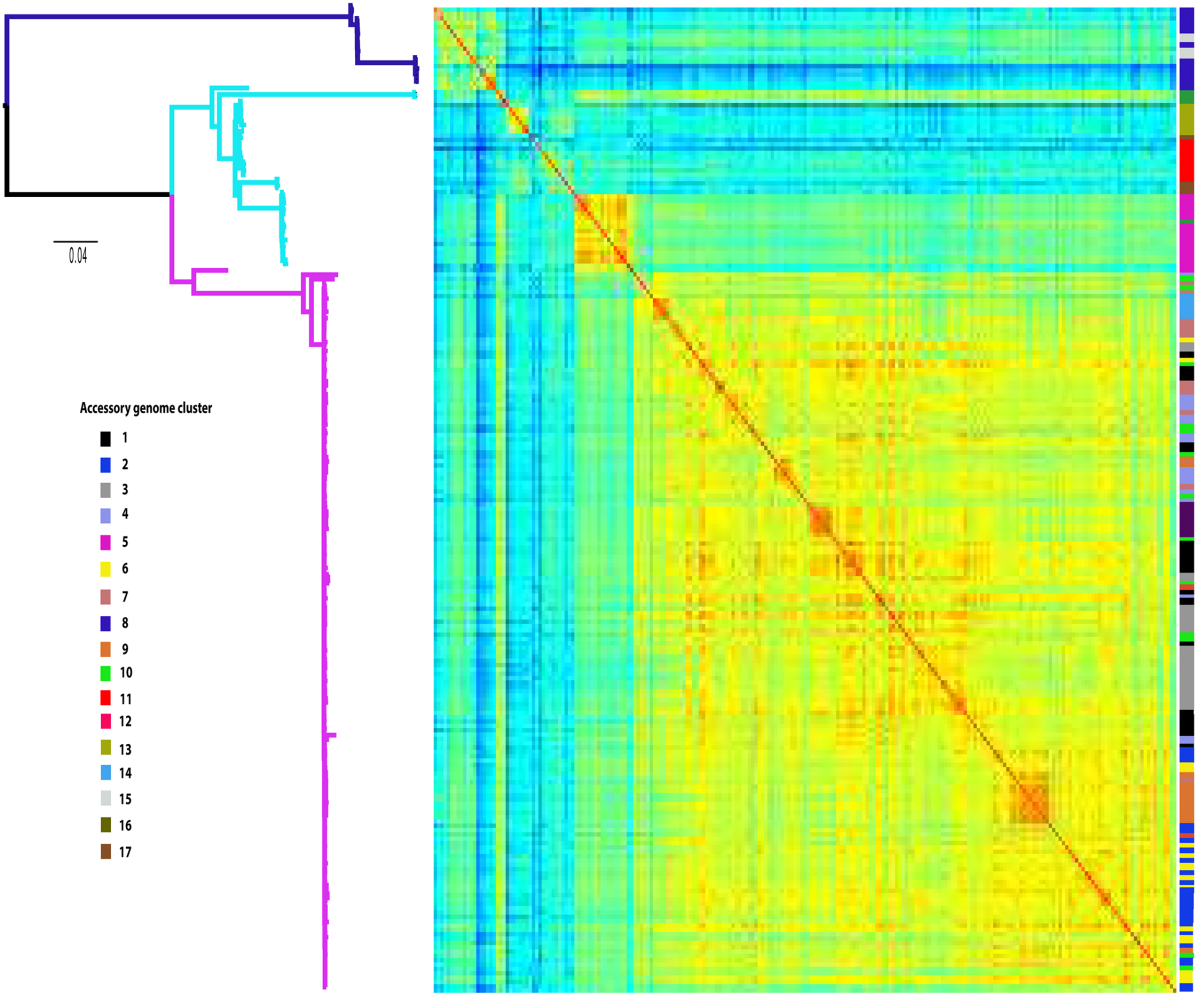
Maximum likelihood phylogeny of the ST131 core genome, with the accessory genome profile overlaid. Clades A, B and C are colour coded by branch (blue, cyan, and magenta respectively). The accessory genome is presented as a heatmap (red = high identity to blue = low identity) of pairwise Spearman correlations of the accessory gene content between each strain, such that warmer colours indicate subsets of isolates with substantially more similar gene content between them than on average between randomly chosen isolates. The colour coding to the right indicates the accessory genome cluster of each strain as determined by Kpax2.

We created a pan-genome matrix for the ST131 data set using LS-BSR (large scale—BLAST score ratio) [21] resulting in a matrix of 11,401 coding sequences, 2,722 of which were present in all isolates and considered as core genes. The remaining 8,679 genes were extracted from the pan-genome to create an accessory genome matrix for all 228 genomes. The genomes were then clustered based on their accessory gene content using the Bayesian clustering analysis tool K-Pax2 [22], resulting in 17 distinct clusters of isolates (Fig 2). Each of these clusters show an association with the type of CTX-M gene carried in agreement with recent data analyzing solely plasmid sequences [23]. We mapped the accessory genome clusters onto the core genome phylogeny (Fig 3, S1 Fig) which showed a high degree of correlation between accessory genome profile and core genome phylogeny in clades A and B, and then multiple accessory genome clusters distributed throughout clade C. Analysis of accessory genome clusters shows that each of these clusters is widely geographically and ecologically distributed indicating multiple circulating subtypes of *E*. *coli* ST131 moving between continents and host species (S2 Fig).

We analysed each accessory genome cluster to identify genes that significantly associated with any given cluster (present in > 80% of genomes in that cluster, and <10% of all other genomes outside the cluster) and which may confer unique biological traits. The only clusters we found with unique genes were clusters 5 and 13, both of which are confined to clade B (S1 Dataset). To confirm our analysis was robust we looked for genes unique to Clades A and C (S1 Dataset) and found the same sets of clade-specific genes previously reported [14]. Therefore whilst clades have unique accessory genes, the accessory genome clusters of isolates we see within clades are as a result of unique combinations of genes circulating in the accessory gene pool. This lends further support to the hypothesis that *E*. *coli* ST131 contains multiple subtypes each of which has arisen as a result of expansion of a successful clone following emergence of a stable accessory gene profile.

Given how robust our accessory genome clustering was, and that *E*. *coli* ST131 has previously been shown to undergo less frequent recombination with *E*. *coli* outside the ST131 lineage [24], we sought to determine whether movement of accessory genes across ST131 strains belonging to different accessory genome clusters was still an ongoing process. The size of the accessory genome matrix presents a computational challenge in such analyses. Additionally, many of the accessory genes may be present in the vast majority of genomes analysed, possibly hindering our detection of any evidence of movement across the population. To address this, we extracted a separate accessory gene matrix containing the rarest accessory genes present in 10% or fewer of the sampled population and analysed their distribution by constructing a bi-partite network of genes versus genomes (S3 Fig, S2 Dataset). The distribution of the rarest genes in the gene pool shows frequent movement across the population indicating that despite having a stable core genome and accessory gene content within distinct clusters, *E*. *coli* ST131 is still capable of undergoing horizontal gene transfer and does so frequently. Identification of these rare genes by blastN comparison against the non-redundant nucleotide database suggested they were primarily phages, transposon, and plasmid genes involved in plasmid mobility and that they are widely disseminated throughout the Enterobacteriacaea.

### Accessory gene content leaves an imprint on core gene regulatory regions

**Fig 4:**
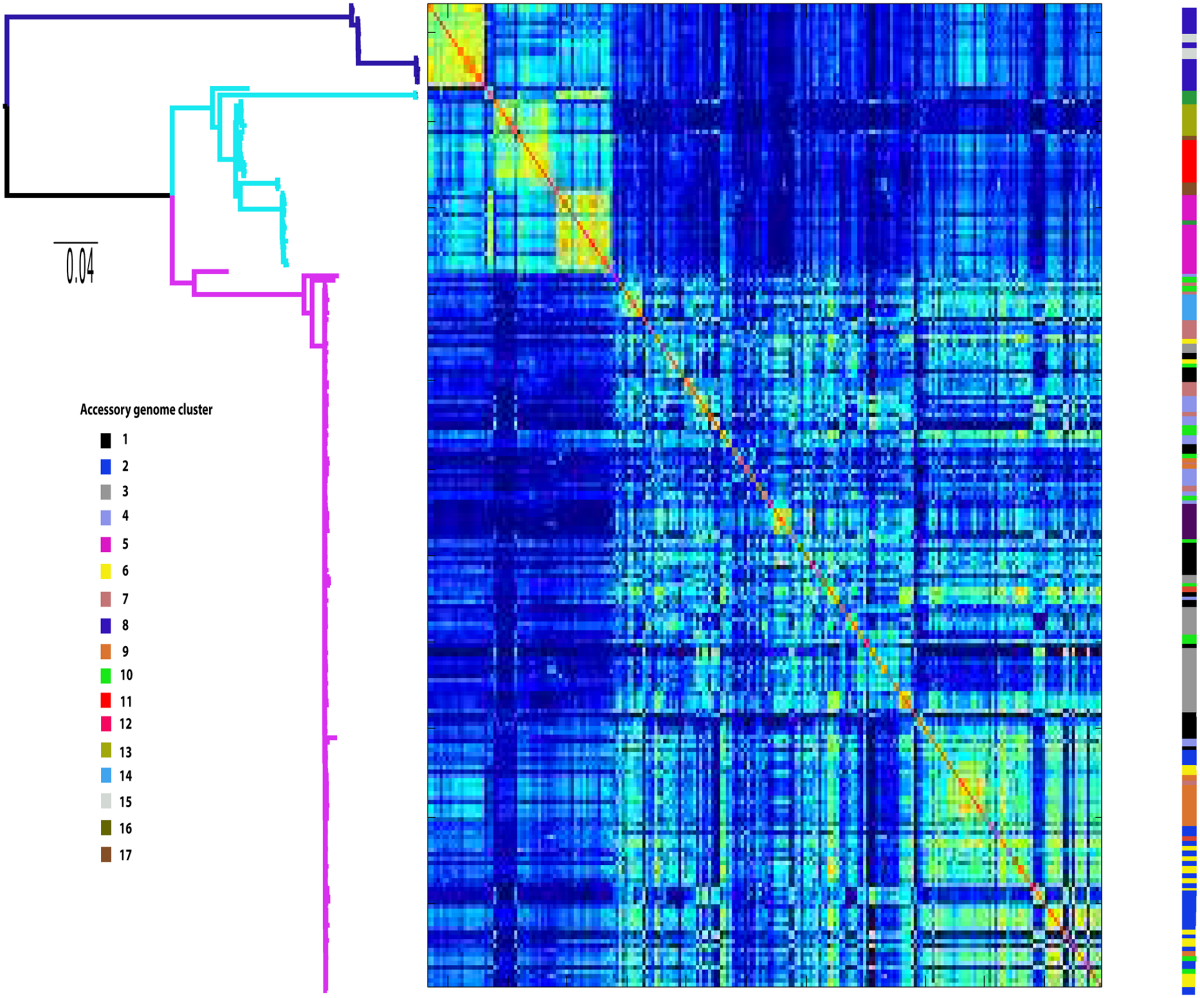
Maximum likelihood phylogeny of the ST131 core genome, with gene regulatory region allele profiles overlaid. Clades A, B and C are colour coded by branch (blue, cyan, and magenta respectively). The gene regulatory region allele profiles are presented as a heatmap (red = high identity to blue = low identity) of pairwise Spearman correlations of the regulatory region alleles between each strain, such that warmer colours indicate subsets of isolates with substantially more similar regulatory region alleles between them than on average between randomly chosen isolates. The colour coding to the right indicates the accessory genome cluster of each strain as determined by Kpax2.

Recent work has highlighted the key role in mobility of gene regulatory regions in determining pathotype specific phenotypes in *E*. *coli* [12]. Moreover, recent experimental evolution studies have highlighted the crucial role of compensatory mutations in regions affecting gene expression in minimising the fitness costs of maintaining resistance plasmids [25,26]. We therefore sought to investigate if the unique accessory genome profiles evident in the ST131 population are associated with changes in gene regulatory regions. *E*. *coli* orthologs were identified and to ensure high conservation among all orthologs within each orthologous group, we filtered out all core clusters in which members showed less than 90% nucleotide identity. Core clusters that potentially included paralogous genes were also filtered out. From this analysis a total of 2,696 regions immediately upstream of CDS core to the ST131 data set were identified. Based on a PRANK alignment of the regions we identified 297 gene regulatory regions exhibiting allelic switching (S3 Dataset). A gene regulatory region allelic profile was then made for the 297 regions for each isolate and mapped onto the core genome phylogeny and accessory genome profile (Fig 4, S4 Fig). We also extracted the sequences of the 297 CDS for which there was allelic switching in gene regulatory regions and created a maximum likelihood phylogeny of the concatenated sequences (S5 Fig). Strikingly, the phylogeny for the gene regulatory region profiles was concordant with that of the accessory genome profiles (Robinson-Foulds distance = 30, 13% incongruence), but not concordant with the core genome phylogeny (Robinson-Foulds distance = 200, 86% incongruence: S5 Fig), indicating that the allele profile of gene regulatory regions in the core genome is directly associated with accessory gene content and is independent of the phylogenetic signal. The incongruence between the core genome alignment and regulatory region alignment is not as a result of differing recombination rates, with the r/m value almost identical for both alignments (0.387 and 0.201 respectively). We performed permutation tests to compare the observed difference in mean correlations of promoter profiles between clades A/B vs between C/B. Clade A/B similarity is higher than B/C (p < 10^−5^). Therefore the gene regulatory region clustering shows that clade A and B display a higher level of identity to each other than to clade C, which suggests that the largest effect on changes in the gene regulatory regions has occurred in response to the prolonged acquisition and maintenance of MDR plasmids that has exclusively occurred in clade C. To further confirm this association we performed a multidimensional scaling analysis of CTX-M gene type and promoter allele profile (S6 Fig) which shows a clear separation of strains with different CTX-M type in combination with the promoter allele profile. The accessory genome clusters are also clearly not independent of the CTX-M type (p = 3.528 x 10^−26^).

The importance of identified gene regulatory region alterations was further emphasized when we performed a pan-genome association analysis on the ST131 data set against 720 complete or draft *E*. *coli* genome sequences downloaded from NCBI (S4 Dataset). The analysis identified a total of 754 loci significantly associated with ST131 (p < 10^-8, S5 Dataset). These were either genes that were significantly more abundant in ST131 than non-ST131 genomes, or genes with a significantly different nucleotide sequence to orthologous genes found in non-ST131 genomes. These included the secondary flagella locus Flag-2, capsular polysaccharide genes previously shown to be ST131 specific [14,27], 292 metabolism associated loci and 91 hypothetical proteins as significantly associated with the ST131 lineage(S7 and S8 Figs, S2 Table). More importantly we identified 87 loci as ST131 unique which were intergenic regions, 64 of which were gene regulatory regions identified as undergoing allele switching in response to accessory gene acquisition (S3 Table). Specifically these were gene regulatory regions which differentiate the clustering of strains between Clade A/B and Clade C.

## Discussion

The data and analytical techniques presented here represent a comprehensive approach to merge fragmented views of bacterial evolution. Combining analysis of the core genome phylogeny, accessory genome profiles and core gene regulatory elements we provide a robust mechanism for understanding the population dynamics of an important MDR lineage of *E*. *coli* and identify significant evolutionary events that have underpinned its emergence as a dominant and successful MDR pathogen. Furthermore, by using strains isolated from non-human reservoirs and under-sampled geographical regions we provide enhanced insight into the complex ecology of an MDR *E*. *coli* lineage beyond the often human-centric approach taken to genomic epidemiological studies of these pathogens.

Our data enables several key conclusions on the ecology of the MDR *E*. *coli* lineage ST131. By including the genomes of companion animal and wild bird isolates into our analysis we were able to show that human, dog, cat, and wild bird isolates can freely move across niches without obvious genomic signals of ecological adaptation and niche segregation. As such our data provides a high-resolution confirmation that the MDR *E*. *coli* lineage ST131 is a host generalist and could be argued to be a zoonotic pathogen. Of particular interest is the inference from our AdaptML analysis concerning a significant ecological jump from a small cluster of predominantly European companion animal isolates in clade A to the rest of the phylogeny, with the only other isolate in the cluster being a human clinical isolate from China which was isolated over 10 years before the European strains. Recent data have shown the importance of fluoroquinolone resistance and virulence gene acquisition to the evolution and emergence of clade C ST131 [23,28]. However, given the fact that ST131 move freely between animal hosts there is an argument that more non-human isolates from all three clades, and more isolates from the understudied clades A and B, would give an even greater level of resolution for understanding the evolutionary events that lead to the creation of the dominant MDR clade C of *E*. *coli* ST131. The power of such an approach is exemplified by the study of human and animal *Salmonella* Typhimurium DT104 isolates at a genome level which showed that human and animal outbreaks were distinct events and not zoonotic infections as previously accepted [29].

The phylogenetic structure of the *E*. *coli* ST131 lineage is well defined [14,15] with all analyses suggesting a single global dissemination of clade C as the driver for the emergence of MDR ST131 as a dominant human clinical isolate [14,15,23,28]. Our study utilises the wealth of information within the accessory gene pool to enhance the resolution of this well-defined population structure. By focusing on the entire accessory gene pool and not just plasmid sequences [23] we show the existence of multiple subtypes of ST131 clade C based on highly congruent accessory gene profiles which often intermingle within the core genome phylogeny. If the levels of admixture observed for the rarest accessory genes throughout the ST131 population held true for all of the accessory gene pool, then over time these clusters would merge and become non-existent, which is inconsistent with our observations. Recent Bayesian dating analysis suggests that clade C diverged around 30–40 years ago and clade B and C from A 60–90 years ago [23,28]. Therefore it seems more likely that the accessory genome clusters of ST131 represent distinct ST131 subtypes which have expanded and disseminated while generally maintaining a defined accessory gene repertoire. The rapid expansion of novel subtypes of *E*. *coli* STs due to gene acquisition has been shown in intestinal pathogenic *E*. *coli* [10,30] but our analysis provides the first indication of this occurring in such a lineage of extra-intestinal pathogenic *E*. *coli*.

Finally, our finding that the alleles of gene regulatory regions of core CDS are concordant with the accessory genome profiles of isolates provides even greater evidence of expansion of multiple subtypes of *E*. *coli* ST131. The importance of the mobility of gene regulatory regions was previously demonstrated in *E*. *coli* [12] and our data set provides the first population level evidence of this phenomenon in *E*. *coli* lineages. Furthermore, our data lend population genomic support to the experimental evolutionary studies suggesting that acquisition and maintenance of MDR plasmids in the absence of antibiotic selection occurs as a result of compensatory mutations that influence gene expression and minimise the fitness costs of the plasmid [25,26]. This is indicated by the fact that clades A and B are closer to each other in terms of gene regulatory profile than to clade C, yet clade B is closer to clade C from a core genome phylogenetic and accessory genome composition perspective. Given that we know the main differences between clade B and clade C to be in the virulence gene profiles and the prevalence of MDR plasmids [14,15,28], this suggests that compensatory mutations in gene regulatory regions as a response to acquisition of MDR plasmids, acts to facilitate the successful emergence of ST131 clade C alongside fluoroquinolone resistance and particular virulence gene alleles [28].

Our work highlights that by combining core, accessory, and gene regulatory region genome analysis it is possible to provide a completely different perspective of the evolution of an extremely well studied and defined bacterial lineage. The enhanced resolution afforded by our approach enabled the generation of an updated hypothesis for the emergence of a globally important MDR *E*. *coli* lineage. According to this hypothesis, a potentially host-restricted group of *E*. *coli* has adapted to become more generalist, resulting in exposure to a more expansive accessory gene pool [31]. The development of fluoroquinolone resistance and selection for important allele variants in virulence genes then occurred to create a lineage adapted to successful human colonization [23,28]. This lineage was subsequently exposed to a number of circulating plasmids, including MDR plasmids, and phages which are acquired and maintained, resulting in compensatory mutations in gene regulatory regions to offset the cost of maintenance. It is also possible that these gene regulatory alleles have been acquired by recombination allowing a more rapid adaptation to the fitness cost, and this is a testable hypothesis that should be focused upon in the immediate future. The above hypothesised evolutionary process has, in summary resulted in successful MDR clones which rapidly disseminate globally and have led to a global healthcare burden. Similar integrated analyses will be of interest in the future to add further resolution to our knowledge of the evolution and emergence of other globally important bacterial pathogens.

## Materials and Methods

### Strains and genomic data

A total of 228 ST131 genomes were analysed in this study (S1 Table). New genomic data was generated for 125 strains with all raw sequence data deposited in Genbank under Bioproject PRJNA295914 or in the ENA, and *de novo* assemblies also deposited in the WGS database as indicated in S1 Table. DNA was extracted using the Sigma GenElute bacterial DNA extraction kit, and evaluated for purity using the Nanodrop system. Sequencing libraries were prepared using the Nextera XT 96-plex library preparation kit and sequenced on the Illumina MiSeq or Illumina HiSeq2500 platforms using V3 sequencing cartridges to provide 2 x 300bp paired-end reads. Genome assemblies for the 125 newly generated genomes were performed using SPAdes v3.6 [32]. The remaining genomes were from previously published studies of ST131 phylogenomics [14, 33] with assembled genomes downloaded from cited repositories [14]. All of the genomes were provided with new annotation using Prokka [34]. The annotated genomes are available from Data dryad (10.5061/dryad.d7d71).

### Core genome analysis

A core genome phylogeny was produced using Parsnp in the Harvest suite of phylogenetic tools [35], which makes an alignment from localised co-linear blocks. The alignment was run with EC958 [36] selected as the reference genome resulting in a core genome alignment of 3.49Mbp. A maximum likelihood phylogeny was inferred from the alignment using RaxML [37] with the GTR-gamma model and 100 bootstrap replicates. The resulting phylogenetic tree was visualised using iTOL which was also used to overlay metadata information [38]. AdaptML was used to infer ecological transitions in the phylogenetic tree [39] using default parameters, with strains divided into habitats of human, companion animal, avian, or livestock.

### Accessory genome analysis

A pan-genome of the ST131 data set was constructed using LS-BSR [21], and a matrix of accessory gene presence/absence for each genome constructed using the filter_BSR_variome.py tool. The resulting accessory genome matrix was used to identify clusters of isolates based on their accessory gene content via Bayesian clustering using Kpax2 [22]. Five independent runs from different starting configurations under the default prior settings and upper bound values for the number of clusters in the interval 30–50 were performed. The optimal clustering was identified using the log posterior scoring function of the method. Average percentages of shared accessory genome content between pairs of strains assigned to the same accessory cluster are shown in S4 Table. The percentages are calculated over the 1850 accessory genes identified by the KPAX2 analysis as significantly discriminatory between the clusters. A heatmap showing pairwise similarities of the accessory genome content was produced in Matlab with the 'image(A)' function, where 'A' is an arbitrary square matrix. A binary alignment based on gene presence-v-absence was created for all strains and a maximum likelihood phylogeny created using the BINCAT model with 100 bootstraps.

The accessory matrix was further filtered so that it included only genes that were present in at least two genomes, but less than 24 genomes (10% of the total). A bipartite graph was constructed from this filtered matrix where each edge had the format [Gene, Genome] (*i*.*e*. an edge connected a gene and a genome). Community structure in this graph was assessed using the Louvain algorithm as implemented in the Gephi software (https://gephi.org/publications/gephi-bastian-feb09.pdf). Communities were collapsed to a single node, consisting of gene and genome nodes, with the size of the nodes reflecting the number of nodes in that community. The layout was achieved using the ForceAtlas2 algorithm implemented in Gephi.

### Gene regulatory regions analysis

Gene regulatory region analysis was performed as previously described [12]. Orthologous genes within the ST131 data set were detected using pairwise reciprocal tblastx best hits. We demanded at least 95% amino acid sequence identity for the region of homology identified by tblastx as high-scoring segment pairs (hsp). In addition, we required that the length of the hsp, excluding gaps, should be longer than 50% of the total query length and that the length of the putative ortholog would not differ by more than 20% from the length of the query sequence. To ensure high conservation among all orthologs within each orthologous group, we used CD-HIT to filter out all core clusters in which some members show less than 90% nucleotide identity. Finally, we filtered out all core clusters that could potentially include paralogous genes (defined as cases in which two different genes were mapped to the same protein).

For each orthologous group, the unaligned regulatory sequences were clustered at the identity level of 80% using CD-HIT. To avoid spurious single-sequence clusters that may arise from sequencing errors, only clusters with at least two sequences were considered. For a regulatory region to be defined as “switched” we further demanded that the the divergence between clusters, calculated based on a PRANK alignment of the regions, would be at least 1.5 times higher than the divergence within clusters. An allelic profile was generated by concatenating, for each strain, the regulatory regions of the 297 identified “switched genes”. A heatmap showing pairwise similarities of the promoter regions between isolates was produced in Matlab with the 'image(A)' function, where 'A' is an arbitrary square matrix.

To obtain a core CDS phylogeny, we used the extract_core_genome.py tool in LS-BSR on our pan-genome matrix to create a core CDS concatenated alignment. We then performed blastN to identify the co-ordinates within the alignment of the CDS for which allele switching had been observed in the gene regulatory region. These regions were extracted from the original core CDS concatenated alignment resulting in a concatenated alignment of the 297 regulatory region switching CDS for all genomes. A maximum likelihood phylogeny was inferred on this alignment using RaxML with the GTR-gamma model and 100 bootstrap replicates.

Statistical analysis of the correlation between CTX-M type and promoter allele profile was performed by standard Chi^2^-test to assess dependence between CTX-M type and accessory clustering. Each strain with a CTX-M plasmid present was categorized according to the CTX-M type and the label of the accessory cluster the strain was assigned to. Independence of the two categorizations was rejected with the p-value equal to 3.528*10^-26. A scatterplot of these data was obtained by a two-dimensional projection of the multi-dimensional scaling of the pairwise Hamming distances of the promoter allele profiles.

### Sequence element enrichment analysis

A total of 720 complete or partial *E*. *coli* genome sequences were downloaded from the NCBI ftp site and selected for use by manual curation (S4 dataset), which involved checking the genome sequences were not ST131 isolates and were not phylogenetic outliers of an *E*. *coli* species tree made from reference genomes. Given the classification into ST131 and non-ST131 strains as the binary variable of interest, we used the alignment-free pan-genomic association analysis introduced in [40] to identify sequence elements that were significantly enriched in the ST131 clade. First, all 948 genome assemblies were scanned with a distributed string mining algorithm for the presence of DNA variation across the strains using k-mers in the length range 10–100. K-mers present in only one or two genomes were excluded from further analysis. The remaining k-mers were tested for positive association with the ST131 clade using either Chi-square tests or logistic regression. In the logistic regression tests the population structure was accounted for by using for each strain the three first coordinate values from multidimensional scaling analysis of the pairwise Hamming distance matrix between strains created from a randomly selected subset of 0.1% of all the k-mers included in the analysis.

## Supporting Information

S1 Fig Maximum likelihood phylogeny of the ST131 core genome, with the accessory genome profile overlaid. Clades A, B and C are colour coded by branch (blue, cyan, and magenta respectively). The accessory genome is presented as gene presence (black) or absence (white). The colour coding to the right indicates the accessory genome cluster of each strain as determined by Kpax2.
(PDF)

S2 Fig Expanded views of regions of the phylogenetic tree of *E*. *coli* ST131. The figure shows the relationship between accessory genome cluster and geographical distribution of 4 selected regions of the whole genome phylogeny. The figure depicts how the relationship between core and accessory genome becomes distorted as the cluster becomes more geographically distributed, likely as a result of increased sharing of rare genes.
(PDF)

S3 Fig Bipartite graph of communities of rare genes against genomes obtained with the Louvain algorithm. Each node is represented by a pie-chart whose segments are coloured according to accessory genome cluster and the sizes are proportional to the number of genes/genomes connected with the particular community.
(PDF)

S4 Fig Maximum likelihood phylogeny of the ST131 core genome, with the alleles of gene regulatory regions overlaid. Clades A, B and C are colour coded by branch (Blue, cyan, and magenta respectively). The alleles are colour coded based on the presence of the presence of differential alleles (white = identical ancestral allele whilst green, blue, red, yellow and magenta represent the presence of minor allele variants of that regulatory region). The colour coding to the right indicates the accessory genome cluster of each strain as determined by Kpax2.
(PDF)

S5 Fig Maximum likelihood phylogeny of the concatenated sequences of the 297 CDS for which gene regulatory region allele switching was observed. (PDF)

S6 Fig Two-dimensional projection of association between CTX-M gene type and promoter allele profile. The plot is based on the multi-dimensional scaling of the pairwise Hamming distances of the promoter allele profiles.
(PDF)

S7 Fig Manhattan skyline plot of k-mers identified as being statistically significantly associated with *E*. *coli* ST131 compared to 720 non-ST131 *E*. *coli* by selected element enrichment analysis. The plot shows the location of all significant k-mers against the reference genome EC958. Selected genetic loci are labelled according to their annotation in the reference JJ11886 genome.
(PDF)

S8 Fig Graphical representation of the function of loci identified as being ST131 unique, or containing ST131 unique alleles, from pangenome GWAS analysis. The Y axis shows that classification of genes based on COG annotation, whilst the x axis shows the number of loci in that functional category containing 1 or more kmer hits from the GWAS analysis.
(PDF)

S1 Table Table of all isolate genomes and individual accession numbers used in this study (XLSX)

S2 Table List of all loci containing kmers significantly associated with *E*. *coli* ST131 compared to non-ST131 *E*. *coli* (XLS)

S3 Table List of 64 loci significantly associated with *E*. *coli* ST131 which have promoter regions undergoing allele switching (XLSX)

S4 Table Average percentages of shared accessory genome content between pairs of strains assigned to the same accessory cluster, for each identified accessory genome cluster (XLS)

S1 Dataset CDS names and nucleotide sequences of accessory genes unique to accessory genome clusters (TXT)

S2 Dataset CDS details and gene accession numbers of the distribution of rarest accessory genes (XLSX)

S3 Dataset Raw data for the regulatory region analysis, including accession number of upstream gene and the relative allele frequencies (XLSX)

S4 Dataset List of *E*. *coli* genomes downloaded from NCBI and used in the SEER analysis (TXT)

S5 Dataset Raw data for the SEER analysis, including the nucleotide sequence of all kmers identified as significant and associated p value. (TXT)
