# Supplementary figures and images for: Combined Analysis of Variation in Core, Accessory and Regulatory Genome Regions Provides a Super-Resolution View into the Evolution of Bacterial Populations
Source: PLoS Genet. 2016 Sep 12;12(9):e1006280. doi: 10.1371/journal.pgen.1006280 (PMC5019451; doi:10.1371/journal.pgen.1006280)

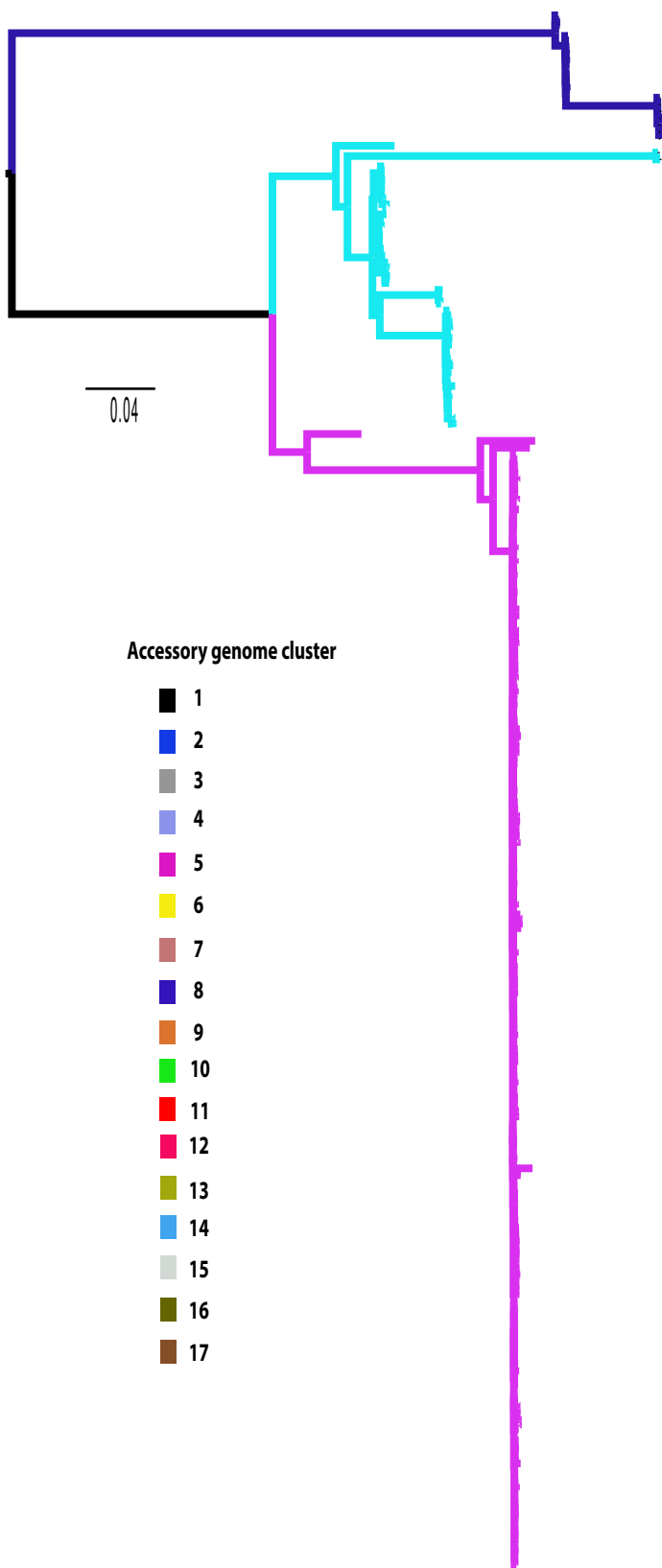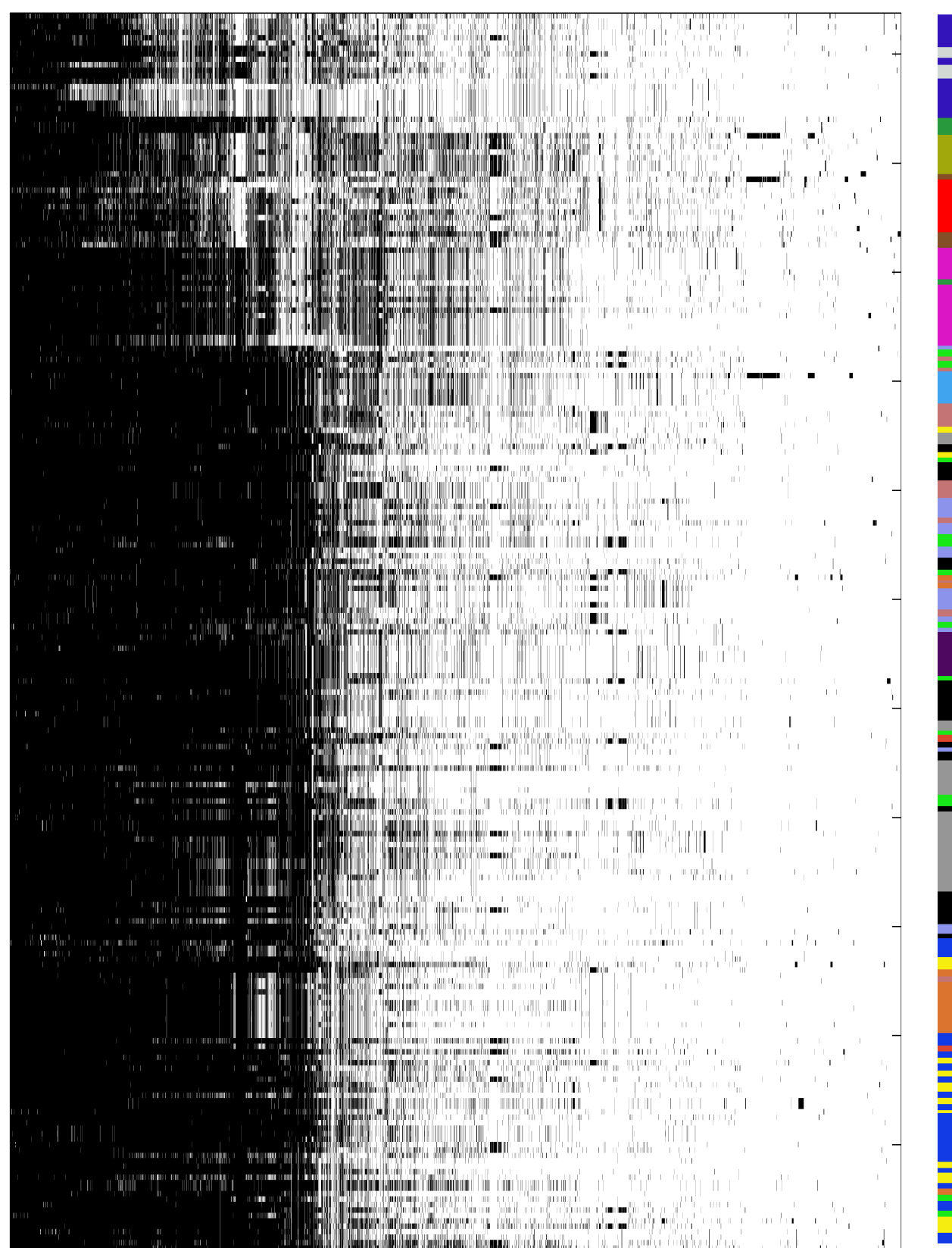

Supplement: S1 Fig — Clades A, B and C are colour coded by branch (blue, cyan, and magenta respectively). The accessory genome is presented as gene presence (black) or absence (white). The colour coding to the right indicates the accessory genome cluster of each strain as determined by Kpax2. (PDF) [file pgen.1006280.s001.pdf]

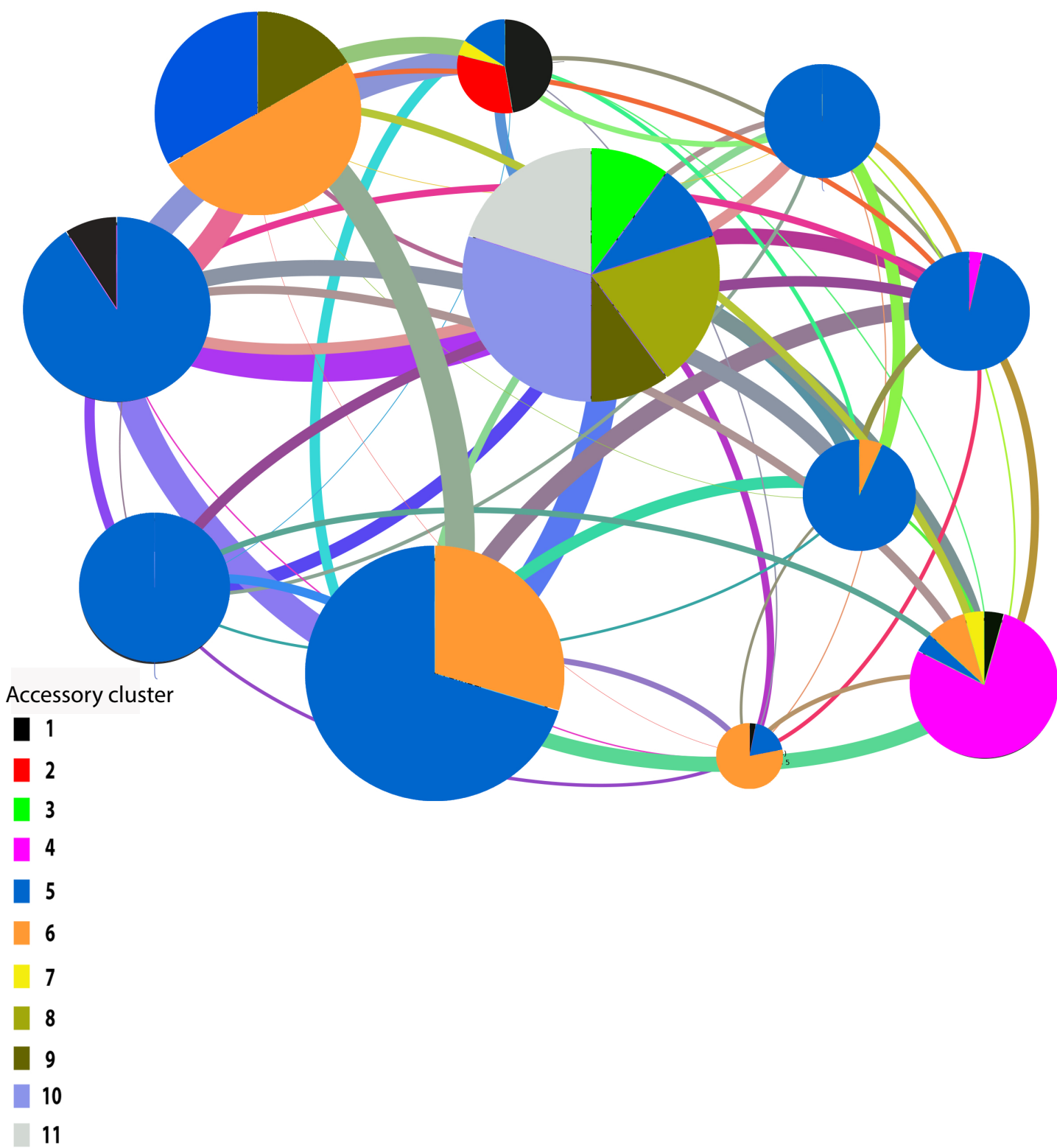

Supplement: S3 Fig — Each node is represented by a pie-chart whose segments are coloured according to accessory genome cluster and the sizes are proportional to the number of genes/genomes connected with the particular community. (PDF) [file pgen.1006280.s003.pdf]

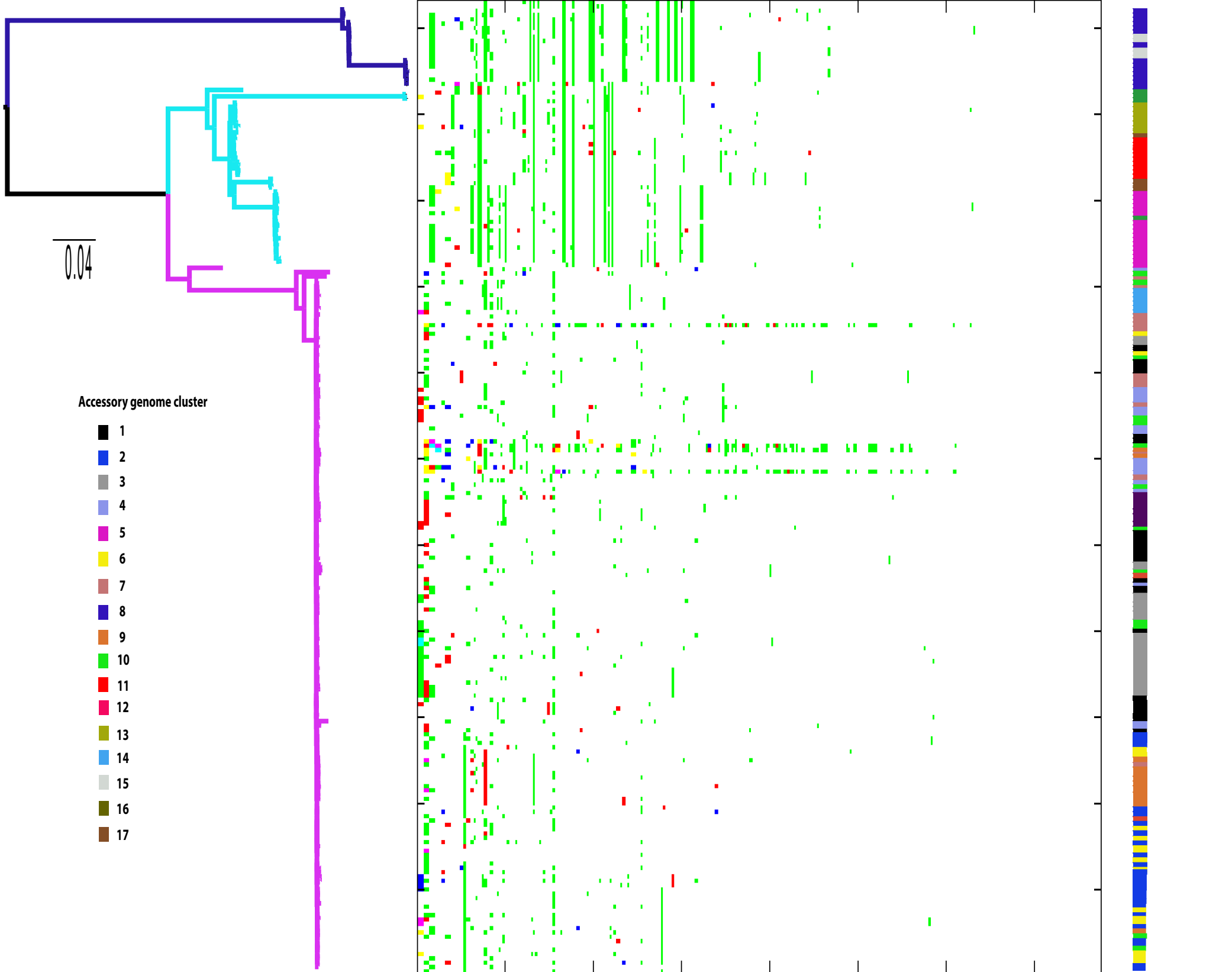

Supplement: S4 Fig — Clades A, B and C are colour coded by branch (Blue, cyan, and magenta respectively). The alleles are colour coded based on the presence of the presence of differential alleles (white = identical ancestral allele whilst green, blue, red, yellow and magenta represent the presence of minor allele variants of that regulatory region). The colour coding to the right indicates the accessory genome cluster of each strain as determined by Kpax2. (PDF) [file pgen.1006280.s004.pdf]

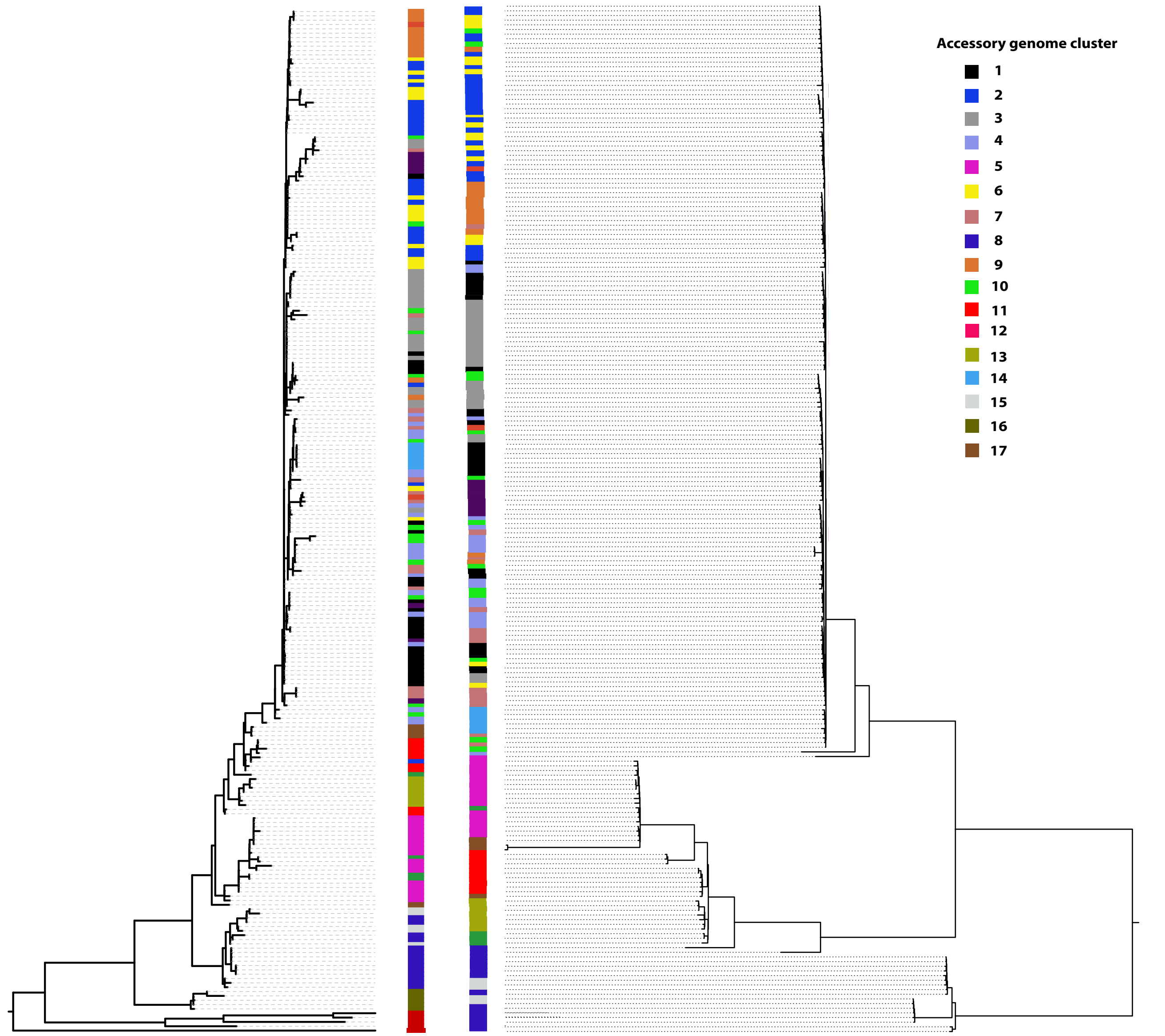

297 promoter phylogeny

297 CDS phylogeny

Robinson-Foulds distance 200 (86% incongruence)

Supplement: S5 Fig — (PDF) [file pgen.1006280.s005.pdf]

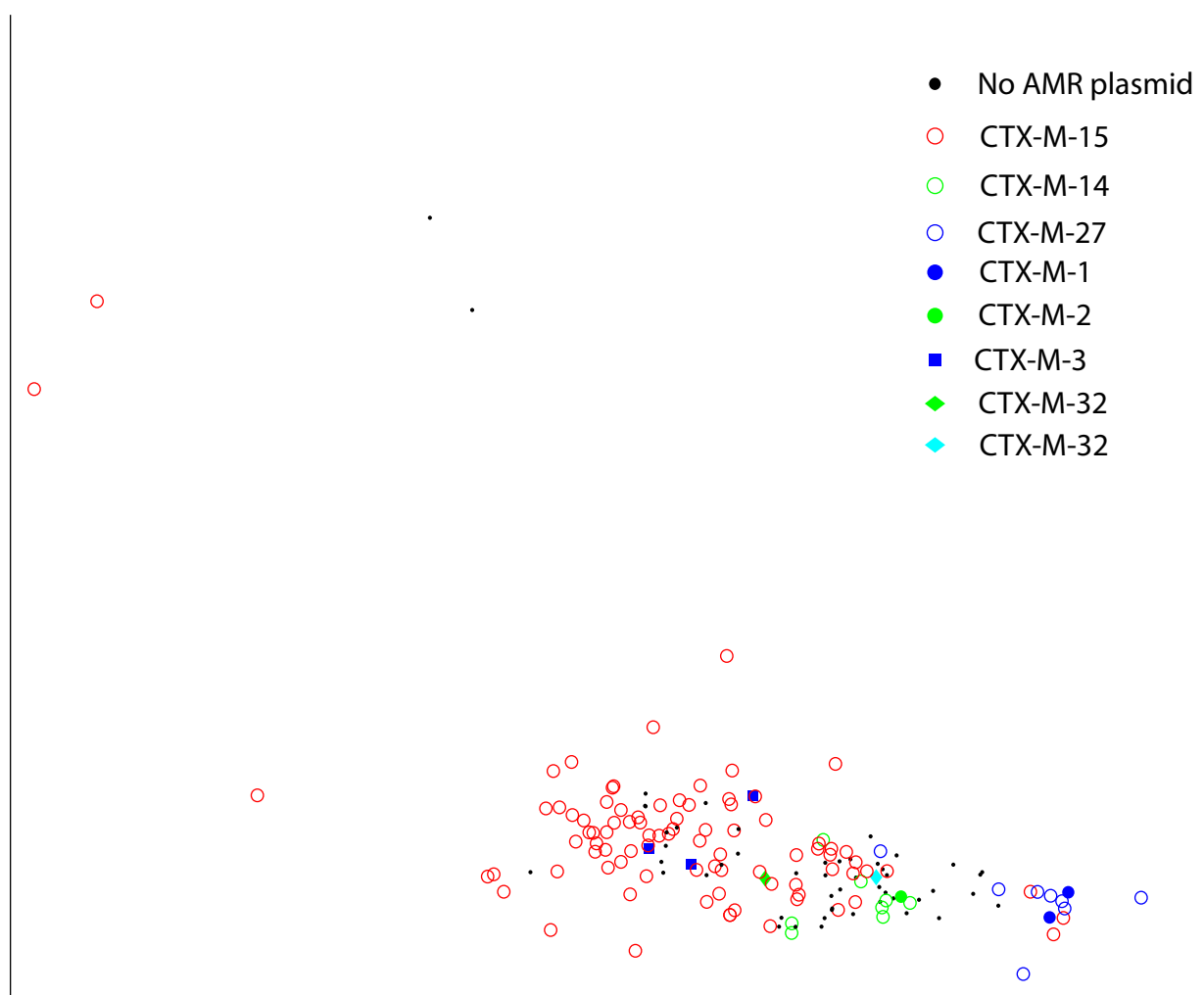

Supplement: S6 Fig — The plot is based on the multi-dimensional scaling of the pairwise Hamming distances of the promoter allele profiles. (PDF) [file pgen.1006280.s006.pdf]

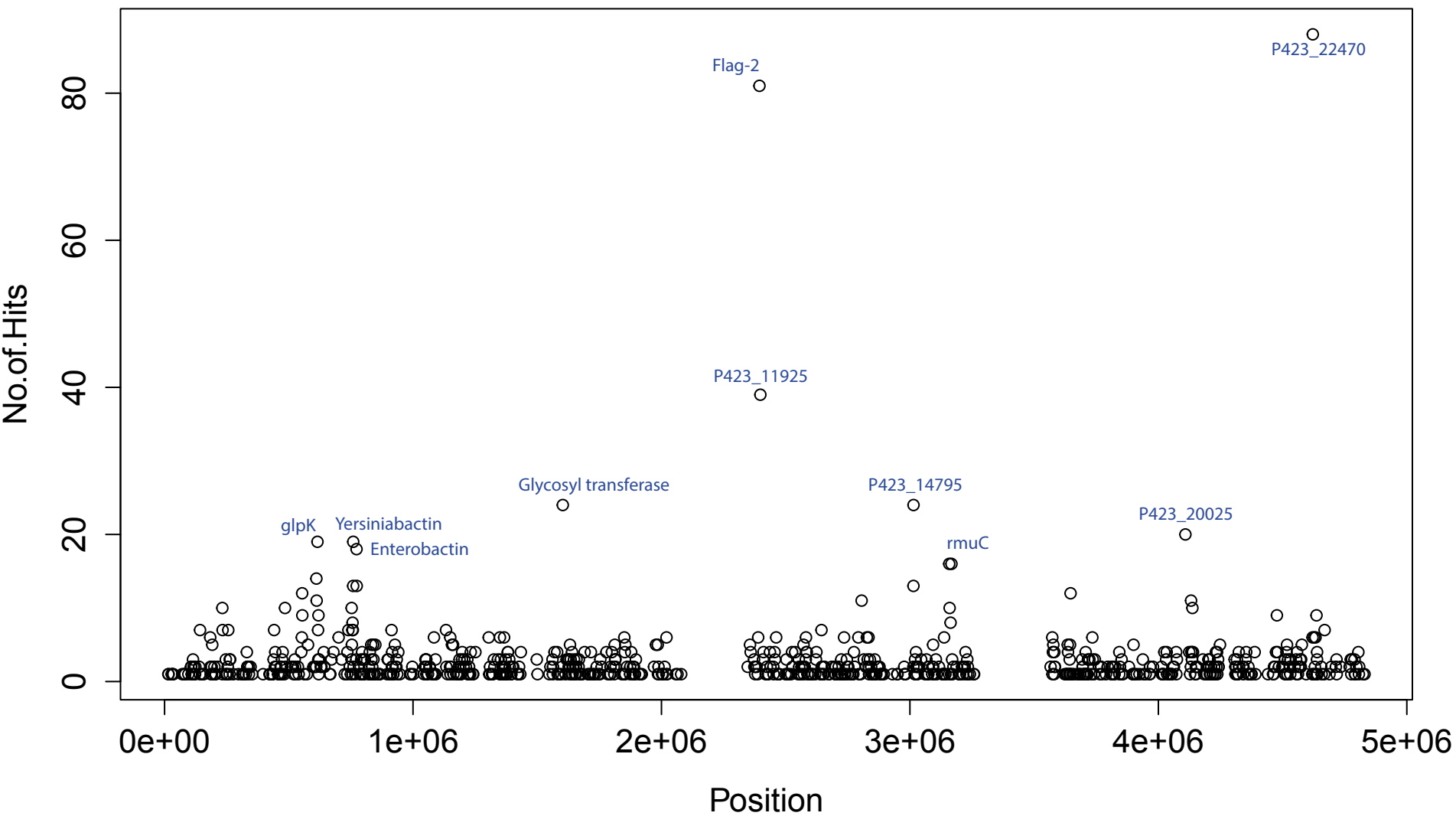

Supplement: S7 Fig — The plot shows the location of all significant k-mers against the reference genome EC958. Selected genetic loci are labelled according to their annotation in the reference JJ11886 genome. (PDF) [file pgen.1006280.s007.pdf]

Number of loci

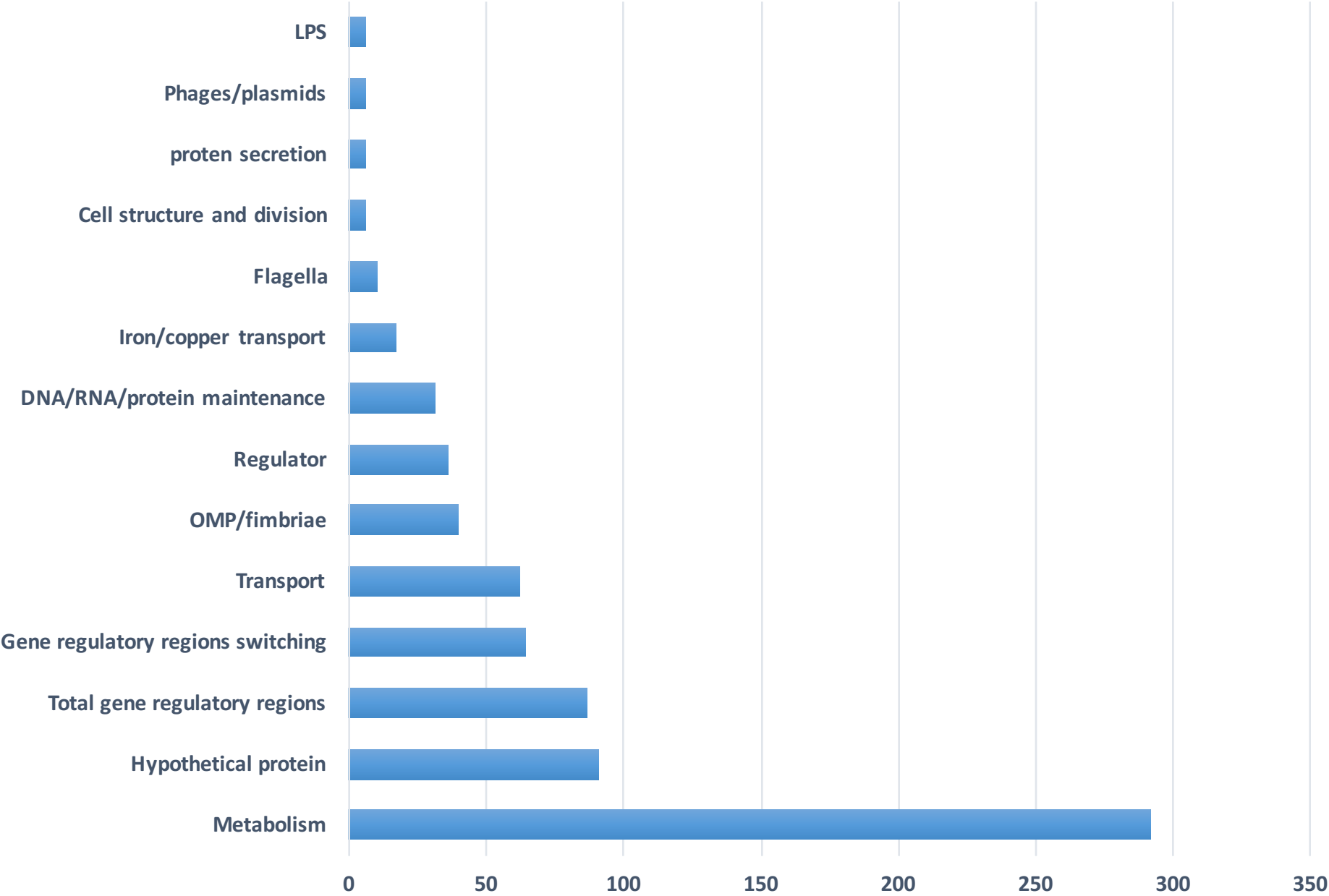

Supplement: S8 Fig — The Y axis shows that classification of genes based on COG annotation, whilst the x axis shows the number of loci in that functional category containing 1 or more kmer hits from the GWAS analysis. (PDF) [file pgen.1006280.s008.pdf]
