# Supplementary material for: Combined Analysis of Variation in Core, Accessory and Regulatory Genome Regions Provides a Super-Resolution View into the Evolution of Bacterial Populations
Source: PLoS Genet. 2016 Sep 12;12(9):e1006280. doi: 10.1371/journal.pgen.1006280 (PMC5019451; doi:10.1371/journal.pgen.1006280)
Supplement: S2 Fig — The figure shows the relationship between accessory genome cluster and geographical distribution of 4 selected regions of the whole genome phylogeny. The figure depicts how the relationship between core and accessory genome becomes distorted as the cluster becomes more geographically distributed, likely as a result of increased sharing of rare genes. (PDF) [file pgen.1006280.s002.pdf]

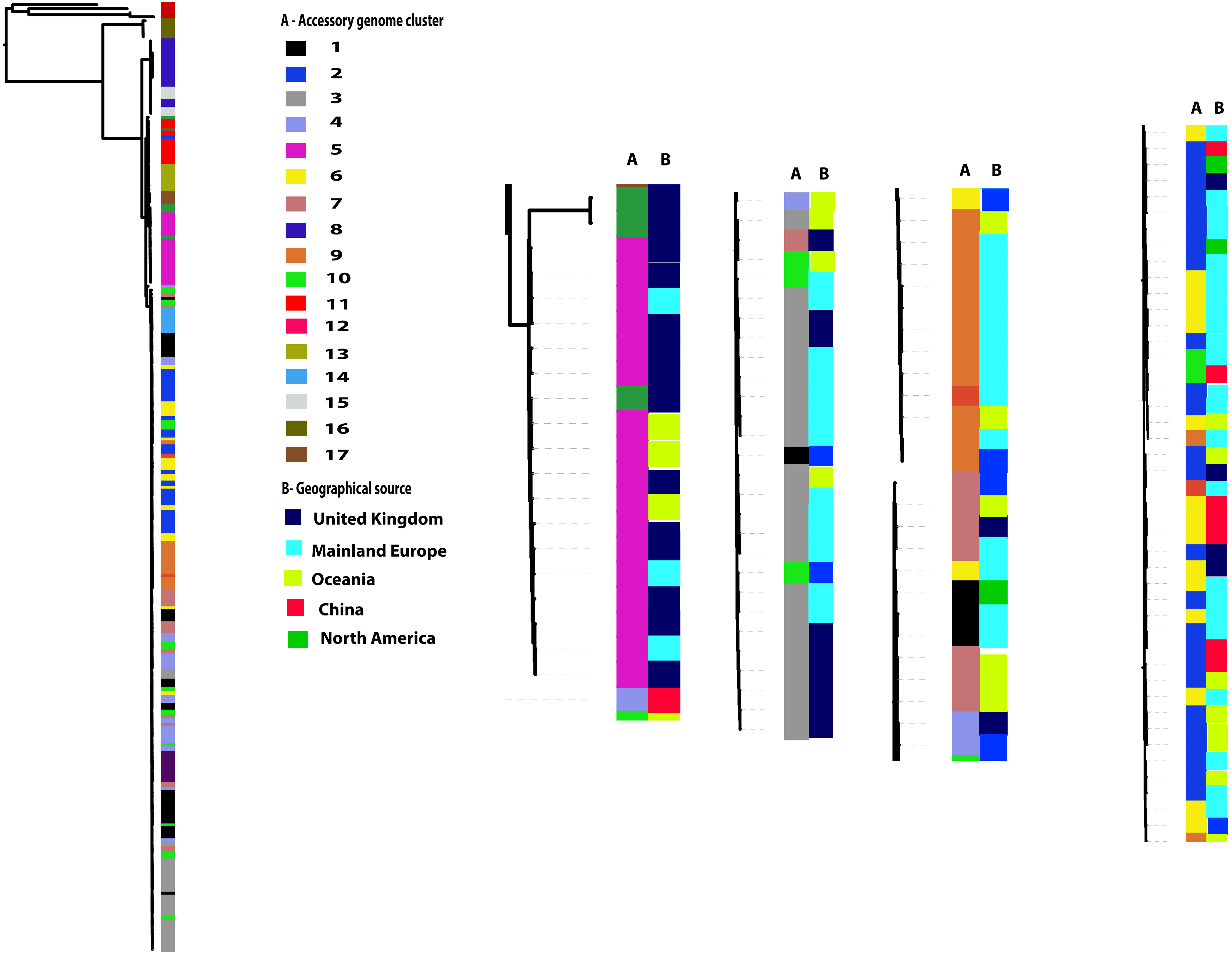
A - Accessory genome cluster
1
2
3
4
5
6
7
8
9
10
11
12
13
14
15
16
17
B- Geographical source
United Kingdom
Mainland Europe
Oceania
China
North America
A
B
A
B
A
B
A
B
